# Placental endovascular extravillous trophoblasts (enEVTs) educate maternal T‐cell differentiation along the maternal‐placental circulation

**DOI:** 10.1111/cpr.12802

**Published:** 2020-04-14

**Authors:** Yeling Ma, Qian Yang, Mengjie Fan, Lanmei Zhang, Yan Gu, Wentong Jia, Zhilang Li, Feiyang Wang, Yu‐xia Li, Jian Wang, Rong Li, Xuan Shao, Yan‐Ling Wang

**Affiliations:** ^1^ State Key Laboratory of Stem Cell and Reproductive Biology Institute of Zoology Chinese Academy of Sciences Beijing China; ^2^ University of Chinese Academy of Sciences Beijing China; ^3^ NHC Key Lab of Reproduction Regulation (Shanghai Institute of Planned Parenthood Research) Fudan University Shanghai China; ^4^ Department of Gynecology and Obstetrics Peking University Third Hospital Beijing China; ^5^ Department of Gynecology and Obstetrics The 306 Hospital of PLA Beijing China; ^6^ Second Hospital Affiliated to Tianjin Medical University Tianjin China

**Keywords:** enEVTs, placental‐maternal circulation, RSA, TGF‐β1, Tregs

## Abstract

**Objectives:**

During human pregnancy, the endothelial cells of the uterine spiral arteries (SPA) are extensively replaced by a subtype of placental trophoblasts, endovascular extravillous trophoblasts (enEVTs), thus establishing a placental‐maternal circulation. On this pathway, foetus‐derived placental villi and enEVTs bath into the maternal blood that perfuses along SPA being not attacked by the maternal lymphocytes. We aimed to reveal the underlying mechanism of such immune tolerance.

**Methods:**

In situ hybridization, immunofluorescence, ELISA and FCM assay were performed to examine TGF‐β1 expression and distribution of regulatory T cells (Tregs) along the placental‐maternal circulation route. The primary enEVTs, interstitial extravillous trophoblasts (iEVTs) and decidual endothelial cells (dECs) were purified by FACS, and their conditioned media were collected to treat naïve CD4^+^ T cells. Treg differentiation was measured by FLOW and CFSE assays.

**Results:**

We found that enEVTs but not iEVTs or dECs actively produced TGF‐β1. The primary enEVTs significantly promoted naïve CD4^+^ T‐cell differentiation into immunosuppressive FOXP3^+^ Tregs, and this effect was dependent on TGF‐β1. In recurrent spontaneous abortion (RSA) patients, an evidently reduced proportion of TGF‐β1–producing enEVTs and their ability to educate Tregs differentiation were observed.

**Conclusions:**

Our findings demonstrate a unique immune‐regulatory characteristic of placental enEVTs to develop immune tolerance along the placental‐maternal circulation. New insights into the pathogenesis of RSA are also suggested.

## 1 INTRODUCTION

The healthy growth of a semi‐allogeneic foetus in uterus requires the adaptive regulation of the maternal immune system to establish an immune‐tolerant environment at the maternal‐foetal interface. Abnormal immune regulation is tightly associated with various pregnancy disorders including recurrent spontaneous abortion (RSA).^1^, ^2^, ^3^, ^4^ RSA affects approximately 1% of the childbearing families.^5^ Around half of RSA cases are derived from unknown reasons, and immune factors have been suggested to be the most important causes in these patients.^6^, ^7^, ^8^, ^9^

Increasing evidence demonstrates the essential effects of T‐cell differentiation in pregnancy maintenance. The immune‐tolerant Th2 and Treg bias during pregnancy are critical to protect the foetus from maternal immune attack.^10^, ^11^, ^12^, ^13^ In vitro and in vivo studies have demonstrated that Tregs sustain immune homoeostasis by suppressing the activation of other leucocytes.^14^ In placental mammals, the generation of Tregs can mitigate the maternal‐foetal conflict, and Treg depletion leads to adverse pregnancy outcomes.^15^ In humans, the proportion of Tregs in peripheral blood and decidua elevates during gestation and returns to non‐pregnant status after delivery.^16^, ^17^, ^18^ The RSA patients exhibit significantly lower numbers of Tregs in both peripheral blood and decidua than healthy pregnant ones.^19^, ^20^, ^21^, ^22^ Transforming growth factor‐beta1 (TGF‐β1) is a key factor that triggers the differentiation of the inducible Tregs through induction of transcription factor FOXP3.^23^, ^24^ In parallel with the change in Treg proportion, peripheral TGF‐β1 level is higher in pregnant women compared with the non‐pregnant individuals,^25^ and its expression is sharply reduced in the peripheral blood and decidua of RSA patients.^26^, ^27^, ^28^, ^29^ It is most likely that TGF‐β1‐regulated differentiation of Tregs critically participates in maintaining a healthy pregnancy.

Physiologically, a placental‐maternal circulatory system is established through the remodelling of uterine spiral arteries (SPA). A subtype of extravillous trophoblasts (EVTs), the endovascular extravillous trophoblasts (enEVTs), invade into SPA and replace the endothelial cells. Another subtype of EVTs, interstitial extravillous trophoblasts (iEVTs), invade decidual stroma and eventually help the loss of vascular smooth muscle cells. The uterine SPA is therefore remodelled into low‐resistance, high‐capacity uteroplacental arteries to ensure the sufficient maternal blood perfusion from SPA to intervillous space (IVS).^30^, ^31^, ^32^, ^33^ It is currently unclear how the foetal‐derived enEVTs in the remodelled SPA and the placenta villous trophoblasts immersing into maternal blood at IVS directly contact the maternal lymphocytes, while do not cause maternal immune rejection along the placental‐maternal circulatory pathway. Considering the localization of enEVTs and the route of maternal blood perfusion, we hypothesize that enEVTs in SPA may essentially educate maternal T‐cell differentiation when maternal blood flows through the remodelled SPA, therefore contributing to local immune tolerance along the placental‐maternal circulation.

To address this hypothesis, we comparatively examined the distribution of Tregs in SPA and IVS from healthy pregnant and RSA women. The TGF‐β1 expression in enEVTs, iEVTs and decidual endothelial cells (dECs) was detected using in situ hybridization, flow cytometry analysis and specific ELISA. The naïve CD4^+^ T cells isolated from healthy women or virgin female mice were treated with the conditioned media from the primarily cultured enEVTs, iEVTs or dECs, respectively, and their differentiation towards functional Tregs was analysed by flow cytometry analysis and carboxyfluorescein succinimidyl ester (CFSE) assays. Our data showed that enEVT is a unique trophoblast subpopulation that can mainly produce TGF‐β1 and induce maternal Treg differentiation. The dysfunction of enEVTs in RSA patients correlates with the failure of their Treg differentiation. Our findings reveal the physiological significance of replacing uterine blood vessel endothelial cells by placental enEVTs from the aspect of immune tolerance and provide a new understanding of the placental pathology of the pregnant disorders such as RSA.

## 2 MATERIALS AND METHODS

### 2.1 Sample collection

The placental villi and decidual tissues from healthy pregnant women (n = 65) or RSA patients (n = 10) at gestational weeks 7‐9 were collected upon therapeutic termination of pregnancy at the 306 Hospital of PLA (Beijing, China) and the Second Hospital Affiliated to Tianjin Medical University (Tianjin, China). The tissues were immersed in iced RPMI‐1640 medium and subjected to cell isolation or fixation within 1 h. Human peripheral blood samples (n = 35) were obtained from healthy non‐pregnant women (aged 25‐35) at Peking University Third Hospital (Beijing, China). The collection of human samples was permitted by the Local Ethical Committees in the Institute of Zoology, Chinese Academy of Sciences, the 306 Hospital of PLA, Peking University Third Hospital, and the Second Hospital Affiliated to Tianjin Medical University.

Recurrent spontaneous abortion was defined according to the criteria of the Practice Committee of the American Society for Reproductive Medicine. In brief, these patients had a history of two or more failed pregnancies for unknown reasons.^34^ Women who manifested an endocrine disorder, foetal chromosomal or congenital abnormalities, uterine anatomical disorders, renal disease or pregnancies conceived by fertility treatment were excluded from this study. All the enrolled patients had arrested foetal development for less than one week before the termination of pregnancy. The clinical characteristics of the pregnant women enrolled in this study are summarized in Table S1.

Mouse spleen tissues were obtained from virgin female SPF C57BL/6 (B6) mice aged 8 weeks (Beijing SPF Biotechnology Co. Ltd.). The experimental procedure was approved by the Animal Welfare and Ethics Committees of the Institute of Zoology, Chinese Academy of Sciences.

### 2.2 Immunohistochemistry

Human decidual tissues were fixed in 4% paraformaldehyde and subjected to routine dehydration and embedding in paraffin wax. The 5‐μm‐thick paraffin sections were subjected to routine rehydration, antigen retrieval and blocking before incubating with a specific antibody against HLA‐G (Abcam, ab52455, 1:500) or NCAM1 (Abcam, ab75813, 1:300). The sections were further incubated with the HRP‐conjugated second antibodies (ZSGB‐BIO, PV‐6001, PV‐6002) and DAB (ZSGB‐BIO, ZLI‐9019) substrate, followed by counterstaining with haematoxylin and mounting. The images were recorded on a light microscope with CCD (DP72, Olympus).

### 2.3 Immunofluorescence

Human decidual or villous tissues were embedded in OCT compound (Sakura Finetek) and frozen sectioned at 8 µm. The frozen sections or the FACS‐isolated cells were briefly fixed in 4% PFA and treated with 0.1% triton, and subjected to the incubation with specific antibodies against HLA‐G (Abcam, ab52455, 1:500), NCAM1 (Abcam, ab75813, 1:300), FOXP3 (Abcam, ab4728, 1:200), Jagged 1 (Abcam, ab7771, 1:200) or cytokeratin 7 (CK7; ZSGB‐BIO, ZM‐0071, 1:200). Binding of the antibody was visualized using FITC‐conjugated or TRITC‐conjugated secondary antibody (ZSGB‐BIO, ZF‐0311, ZF‐0313, 1:100), and cell nuclei were stained with 4,6‐diamidino‐2‐phenylindole (DAPI; Sigma, 28718‐90‐3). The results were recorded using a Zeiss LSM780 confocal system (Zeiss) and processed with ZEN 2012 software (Zeiss). To quantify the number of Tregs in a unit area of SPA and IVS, the regions of SPA or IVS in one image view were lined out, and the pixels were counted and transformed to area (mm^2^) by Image‐Pro Plus 6.0 (Media Cybernetics). At least three random views were analysed for each section, and 10 cases each from healthy pregnancy or RSA group were randomly selected for analysis.

### 2.4 In situ hybridization

TGF‐β1–specific riboprobe was designed as nucleotides 461‐1362 of Homo sapiens TGF‐β1 mRNA. The probe was in vitro transcribed from human cDNA templates using forward primer: AAGACTTTTCCCCAGACCTCG and reverse primer: TGTATTTCTGGTACAGCTCCACG. Digoxin‐labelled riboprobe was synthesized according to the manufacturer's protocol (Roche, 11175025910). Human decidual tissues were embedded in OCT compound immediately after abortion surgery, and frozen sections at 10 µm were briefly fixed in 4% PFA, hybridized with the riboprobe overnight at 55°C. The slides were incubated with AP‐conjugated anti‐digoxin antibody (Roche, 11093274910) and visualized with BCIP/NBT (Promega, 30542801, 30395402) as substrate. Following the in situ hybridization for TGF‐β1, the slides were further subjected to the incubation with antibodies against HLA‐G (Abcam, ab52455, 1:500) or CD31 (Abcam, ab28364, 1:100). Signals were visualized by the incubation with HRP‐labelled secondary antibody and DAB substrate. Fast red staining was performed to show cell nuclei before mounting the slides.

### 2.5 Cell isolation, culture and treatment

Freshly collected human decidual tissues were trimmed into 1‐mm^3^ pieces by GentelMACS Dissociator (Miltenyi Biotec, 130‐093‐235) and digested with 1.0 mg/mL type IV collagenase (Gibco, 9001121) and 10 U/mL type I DNase (Sigma, DN25). The cells were incubated overnight in RPMI‐1640 (Gibco, 31800‐022) supplemented with 10% FBS (Gibco, 10270‐106), and the adherent cells were collected and stained with eFluor 506‐viability dye (eBioscience, 65‐0866‐14), PE‐labelled anti‐HLA‐G (eBioscience, 12‐9957‐42) and APC‐labelled anti‐NCAM1 (BioLegend, 318310), or APC‐labelled anti‐NCAM1 (BioLegend, 318310) and PE‐labelled anti‐CD31 (ebioscience, 12‐0319‐41). Flow sorting with MoFlo XDP (Beckman Coulter, Inc) was further performed to purify enEVTs, iEVTs and dECs, which were labelled as HLA‐G^+^NCAM1^+^, HLA‐G^+^NCAM1^−^ and NCAM1^−^CD31^+^, respectively. The sorted cells were cultured in 96‐well plate (Corning, 3599) at 9000 cells per well in RPMI‐1640 media supplemented with 10% FBS. The conditioned media (enEVT‐CM, iEVT‐CM and dEC‐CM) were harvested after 24 h of culture. Decidual tissues from at least three cases were pooled to isolate primary cells, and at least three batches of experiments were independently repeated.

Naïve CD4^+^ T cells were isolated from human peripheral blood or mouse spleen. In brief, anti‐coagulant peripheral blood from 5 healthy non‐pregnant women was pooled (20 mL in total) and subjected to Ficoll‐Paque Plus (GE Healthcare, 17‐1440‐02) separation and naïve CD4^+^ T‐cell negative selection microbeads (Miltenyi, 130‐094‐131) isolation according to the manufacturer's instruction. In total, 7 batches of human naïve CD4^+^ T cells were isolated from the 35 enrolled women.

The spleens from female virgin B6 mice (Beijing SPF Biotechnology Co., Ltd.) were ground and centrifuged, followed by erythrocyte lysis (BD Biosciences, 555899). The lymphocytes were stained with Pacific blue‐labelled anti‐CD4 (BioLegend, 100428), APC‐labelled anti‐CD62L (eBioscience, 17‐0621‐82) and PerCP‐Cyanine5.5‐labelled anti‐CD44 (eBioscience, 45‐0441‐82) antibodies, and subjected to flow cytometry sorting (Beckman Coulter) to obtain CD4^+^ CD44^−^ CD62L^+^ naïve T cells.

The naïve CD4^+^ T cells were cultured in 96‐well plate at 10^5^ cells/well and activated with 2 μg/mL anti‐CD3 (eBioscience, 16‐0037‐85, 16‐0031‐81) and anti‐CD28 (eBioscience, 16‐0289‐81, 16‐0281‐81), followed by treatment with 50% enEVT‐CM, iEVT‐CM or dEC‐CM for 3 days, together with or without 20 μg/mL blocking antibody against TGF‐β1 (RD System, MAB240‐100) or mouse anti‐IgG (Abmart, B30010M).

### 2.6 Flow cytometry

Flow cytometry for TGF‐β1–expressing cells or Tregs was performed in CytoFLEX (Beckman Coulter, Inc) using the following antibodies or kits: PE‐labelled anti‐HLA‐G (eBioscience, 12‐9957‐42), APC‐labelled anti‐NCAM1 (BioLegend, 318310), FITC‐labelled anti‐TGF‐β1 (BioLegend, 349606), human Regulatory T Cell Staining Kit (eBioscience, 88‐8995) and Mouse Regulatory T Cell Staining Kit (eBioscience, 88‐8111‐40), according to the manufacturer's instructions. Data were analysed using CytExpert (Beckman Coulter, Inc).

### 2.7 ELISA for TGF‐β1

Levels of TGF‐β1 secretion in cell supernatants were analysed by using a sandwich ELISA according to the manufacturer's instruction (ProteinTech, KE00002). In brief, the conditioned media from cultured cells were pre‐incubated with 1 N HCl followed by neutralization with 1 N NaOH and subjected to sandwich ELISA. The levels of TGF‐β1 were determined based on the standard curve.

### 2.8 CFSE assay

CFSE assay was carried out according to the manufacturer's instruction (Invitrogen, C34570). In brief, mouse CD4^+^ CD25^−^ T cells were labelled with 2.5 μM cell division tracking dye CFSE and seeded at 3 × 10^4^ per well in 96‐well plates, which were pre‐coated with 2 μg/mL anti‐CD3 (eBioscience, 16‐0031‐81) and anti‐CD28 (eBioscience, 16‐0281‐81). CD4^+^ CD25^‐^ T cells were cultured alone or co‐cultured with enEVT‐primed Tregs at a ratio of 2:1 for 4 days. The cells were then subjected to flow cytometry analysis for CD4^+^ CD25^‐^ T‐cell proliferation in CytoFLEX (Beckman Coulter, Inc), and the data were analysed with CytExpert (Beckman Coulter, Inc).

### 2.9 Statistical analysis

The statistical analyses were performed with GraphPad Prism version 5.01 (GraphPad Software) and SPSS 18.0 software package (IBM). Data are shown as mean ± SD according to at least three independently repeated experiments. Statistical comparison was performed by using independent sample *t* test or unpaired one‐way analysis of variance (ANOVA) with correction by the Tukey method. The *P* values of <.05 were considered statistically significant.

## 3 RESULTS

### 3.1 Distribution pattern of Tregs along the placental‐maternal circulation pathway

**FIGURE 1:**
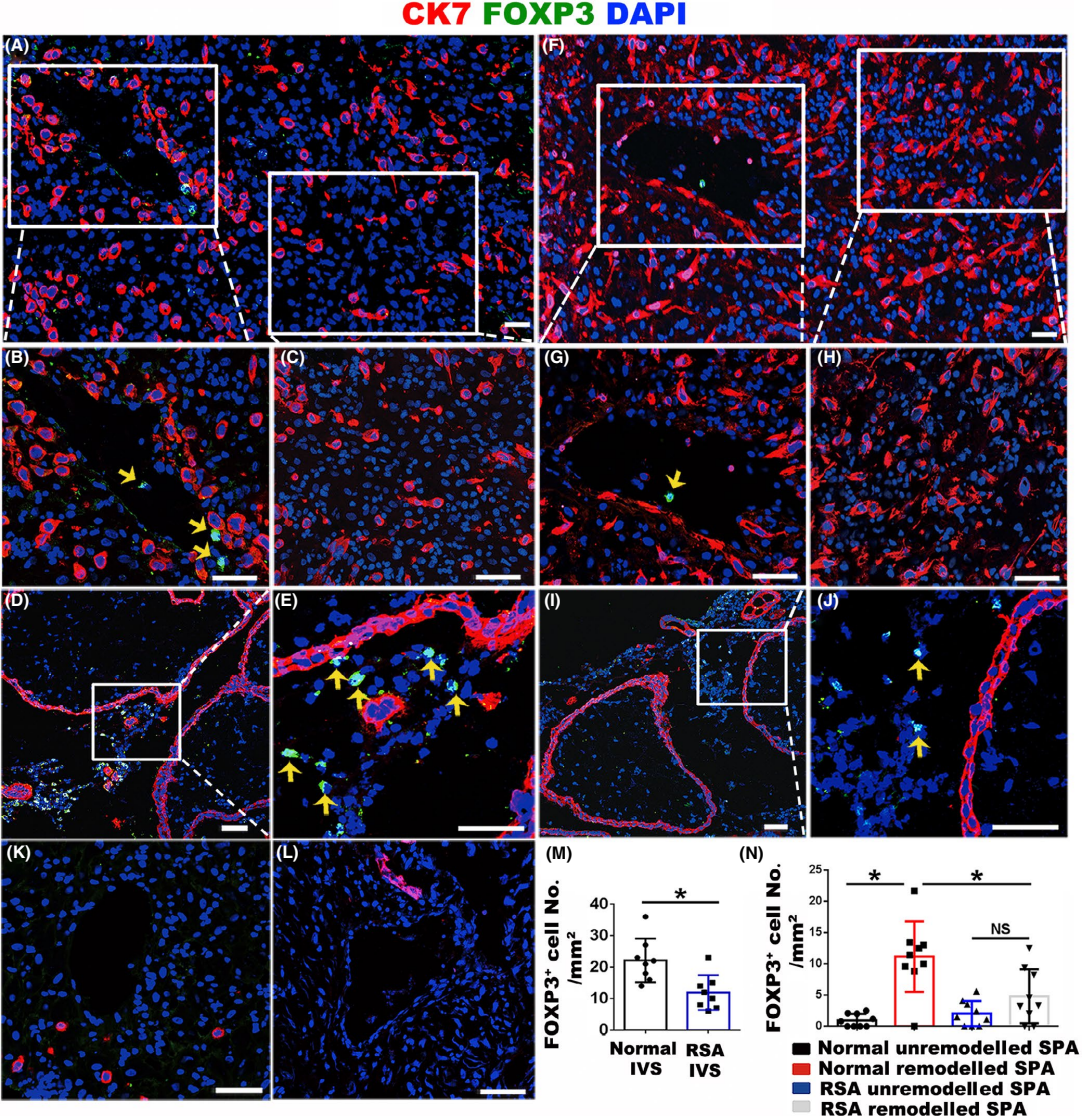
Distribution and proportion of Tregs at the maternal‐foetal interface in healthy and RSA pregnancies at gestational weeks 7‐8. A, Immunofluorescent staining of CK7 (red) and FOXP3 (green) in normal pregnant decidua. B, C, Enlargement of the areas as indicated in panel a, showing remodelled SPA (B) and the area nearby the remodelled SPA (C). D, E, Immunofluorescent staining of CK7 (red) and FOXP3 (green) in placental villi of normal pregnancy and the enlargement of the IVS area are shown in panel E. F, Immunofluorescent staining of CK7 (red) and FOXP3 (green) in RSA decidua. G, H, Enlargement of the areas as indicated in panel F, showing remodelled SPA (G) and the area nearby the remodelled SPA (H). I, J, Immunofluorescent staining of CK7 (red) and FOXP3 (green) in placental villi of RSA pregnancy and the enlargement of the IVS area are shown in panel J. K, L, Immunofluorescent staining of CK7 (red) and FOXP3 (green) in unremodelled SPA of normal pregnancy (K) and RSA pregnancy (L). M, N, The statistical analysis of FOXP3^+^ Treg number in a unit area of IVS (M) and SPA (N) in normal and RSA pregnancies. Three random views from each case were counted, and results from 3 pairs of normal and RSA cases were statistically analysed using ANOVA. Data are presented as mean ± SD. *P < .05. Scale bars indicate 100 μm. Yellow arrows indicate FOXP3^+^ Tregs

To illustrate the distribution of Tregs at the maternal‐foetal interface, especially along the placental‐maternal circulation pathway, we performed immunofluorescence staining for CK7 and FOXP3 in human decidual tissues at early pregnancy, which specifically marked trophoblasts and Tregs, respectively. In typical pregnant cases (Figure 1A‐E), FOXP3^+^ Tregs existed in the lumen of the remodelled SPA (Figure 1A,B) and the IVS area (Figure 1D,E). The area of SPA or IVS in one view was measured by Image‐Pro, and the number of Tregs in unit area of SPA and IVS was statistically quantified. Data revealed that in RSA decidua (Figure 1F,J), the proportion of FOXP3^+^ Tregs in the lumen of remodelled SPA (Figure 1F,G) and IVS (Figure 1I,J) were significantly lower than that in normal pregnancy decidua (Figure 1M,N). Few Tregs were found in the non‐remodelled SPA, either in normal (Figure 1K,N) or in RSA (Figure 1L,N) pregnancy. In addition, very few FOXP3^+^ Tregs were observed in the decidual stroma, where iEVTs were clustered (Figure 1C,H).

### 3.2 enEVTs, but not iEVTs or dECs, could specifically produce TGF‐β1

The above data showed specific distribution of FOXP3^+^ Tregs along the route of maternal blood perfusion. It is well known that TGF‐β1 is the master regulator that triggers Treg differentiation.^23^, ^24^ To address whether the FOXP3^+^ Tregs in SPA and RSA were potentially induced by any TGF‐β1–producing cells at the placental‐maternal circulation, we first detected the expression of TGF‐β1 in decidual tissues.

**FIGURE 2:**
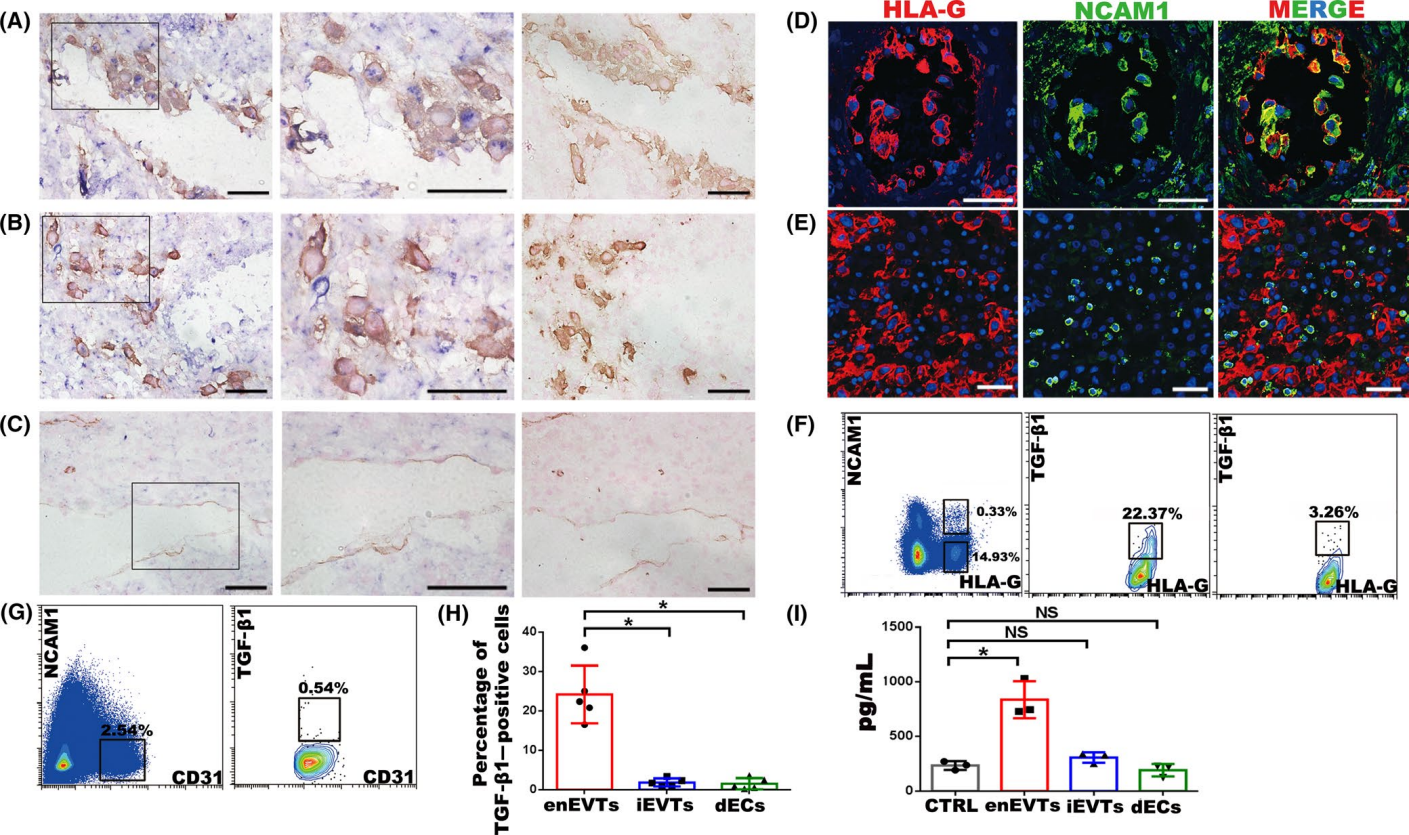
Identification of TGF‐β1–producing cells at the maternal‐foetal interface in normal pregnancy at gestational weeks 7‐9. A‐C, In situ hybridization of TGF‐β1 (blue) and immunohistochemistry staining of HLA‐G (yellow in A and B) or CD31 (yellow in C) in normal pregnant decidua. Middle panels are enlargement of the rectangular areas in left panels, showing the enEVTs in remodelled SPA (A), iEVTs in the area nearby the remodelled SPA (B) and dECs in unremodelled SPA (C). Right panels are negative control of in situ hybridization, with the immunohistochemistry signals of HLA‐G (A, B) or CD31 (C). D, E, Immunofluorescence of HLA‐G (red) and NCAM1 (green) in remodelled SPA (D) and the area nearby remodelled SPA (E). F, Flow cytometry of TGF‐β1 expression in EVTs of normal pregnancy. Left panel, FACS isolation of enEVTs and iEVTs with antibodies against HLA‐G and NCAM1. Middle panel, flow cytometry of TGF‐β1–positive enEVTs that are gated from the left panel as HLA‐G^+^NCAM1^+^. Right panel: flow cytometry analysis of TGF‐β1–positive iEVTs that were gated from the left panel as HLA‐G^+^NCAM1^−^. G, FACS isolation of CD31^+^NCAM1^−^ dECs in normal pregnancy (left panel), and flow cytometry of TGF‐β1–positive dECs gated from the left panel as CD31^+^NCAM1^−^ (right panel). H, The statistical analysis of TGF‐β1–positive primary cells based on the results from 5 normal pregnant cases. I, ELISA for TGF‐β1 in supernatants of the FACS‐sorted enEVTs, iEVTs and dECs (n = 3). The foetal bovine serum in cell‐free media also contains TGF‐β1, so the cell‐free media was set as a control group (CTRL). Data are presented as mean ± SD, and comparison between groups was performed with Student's *t* test. *P < .05. NS, no significance. Scale bars indicate 100 μm

In normal decidua at early pregnancy, in situ hybridization for TGF‐β1 and immunohistochemistry for an EVT marker, HLA‐G, were conducted. We found strong positive signals for TGF‐β1 in the majority of enEVTs in the remodelled SPA (Figure 2A), while few signals in iEVTs (Figure 2B). The dECs that were marked by CD31 exhibited also scarce TGF‐β1 signal (Figure 2C).

We then isolated the primary enEVTs, iEVTs and dECs from normal decidual tissues at early pregnancy to measure TGF‐β1 production. So far, the knowledge of enEVT properties is limited, and the known markers include HLA‐G, NCAM1 and Jagged1.^35^, ^36^, ^37^, ^38^ Our immunofluorescence and immunohistochemistry in human decidual tissues clearly showed that enEVTs were positive for both HLA‐G and NCAM1 (Figure 2D, Figure S1A), while iEVTs were positive for HLA‐G but negative for NCAM1 (Figure 2E). Therefore, FACS was performed to obtain NCAM1^+^ HLA‐G^+^ enEVTs and NCAM1^‐^ HLA‐G^+^ iEVTs from human decidual tissues, and their proportions were around 0.3% and 15% of whole decidual cells, respectively (Figure 2F, Figure S2). Immunofluorescence for Jagged1 and CK7 further proved the high purity of these isolated cells (Figure S1B). dECs were isolated as NCAM1^‐^ CD31^+^ cells in the decidual tissues (Figure 2G). In these primary cells, flow cytometry analysis of TGF‐β1 revealed that approximately 22% of enEVTs, 4% of iEVTs (Figure 2F) and 1% of dECs (Figure 2G) expressed TGF‐β1. Statistical analysis showed that the percentage of TGF‐β1–positive cells in enEVTs was significantly higher than that in iEVTs and dECs (Figure 2H).

The isolated primary cells were cultured for 24 hours, and TGF‐β1 concentration in the supernatants/conditioned media was measured by using specific ELISA. The cell‐free medium was used as the negative control (NC). The concentration of TGF‐β1 in iEVTs or dECs was comparable to NC, while that in enEVTs was approximately fourfold higher of NC (Figure 2I).

**FIGURE 3:**
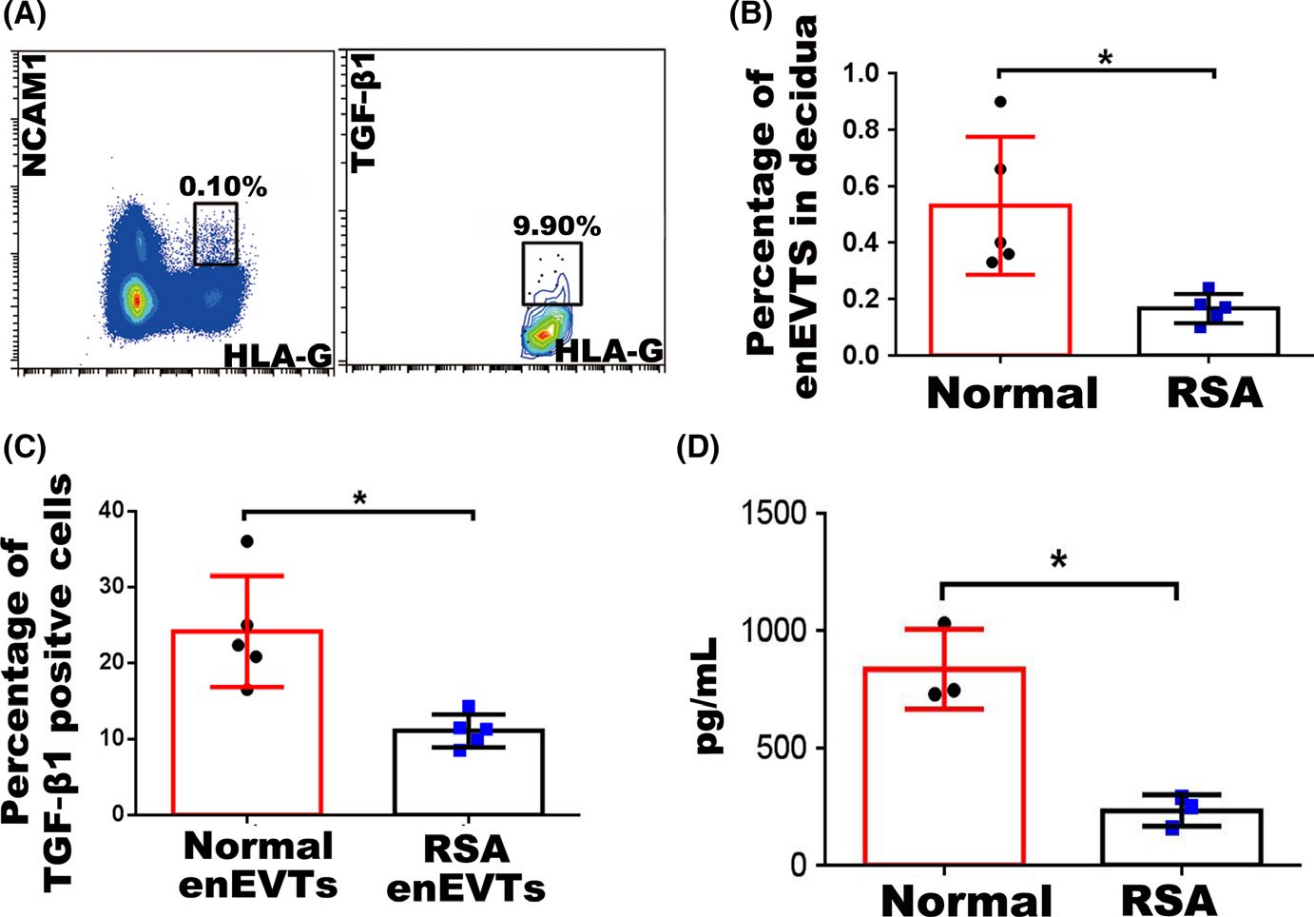
The number of enEVTs and TGF‐β1–producing enEVTs in RSA pregnancy. A, Flow cytometry of enEVTs with antibodies against HLA‐G and NCAM1 in RSA pregnancy (left panel). Flow cytometry of TGF‐β1–positive enEVTs that are gated from the left panel as HLA‐G^+^NCAM1^+^ in RSA pregnancy (right panel). B, The statistical analysis of the number of enEVTs in normal (n = 5) and RSA (n = 5) pregnancy. C, The statistical analysis of the proportion of TGF‐β1–producing enEVTs in normal (n = 5) and RSA (n = 5) pregnancy. D, ELISA for TGF‐β1 in supernatants of the normal enEVTs (n = 3) and RSA enEVTs (n = 3). Data are presented as mean ± SD, and comparison between groups was performed with Student's *t* test. *, *P* < .05

In RSA decidua, flow cytometry showed that the NCAM1^+^ HLA‐G^+^ enEVTs accounted for <0.1% of the decidual cells (Figure 3A). Furthermore, only 9.9% of these enEVTs were TGF‐β1 positive (Figure 3A). Both the number of enEVTs and the proportion of TGF‐β1–producing enEVTs in RSA decidua were significantly lower than those in normal decidua (Figure 3B,C). Results of ELISA showed the TGF‐β1 secretion in the RSA enEVTs was significantly reduced compared with normal enEVTs (Figure 3D).

The data revealed that enEVTs, but not iEVTs or dECs, were the unique cells being capable of producing TGF‐β1, and their number, as well as TGF‐β1 production, decreased remarkably in RSA pregnancy.

### 3.3 enEVTs utilized TGF‐β1 to promote functional Treg differentiation

To explore whether enEVTs are efficient in priming Treg differentiation, we cultured the primary enEVTs for 24 hours and collected their conditioned media (enEVT‐CM) to treat naïve CD4^+^ T cells.

**FIGURE 4:**
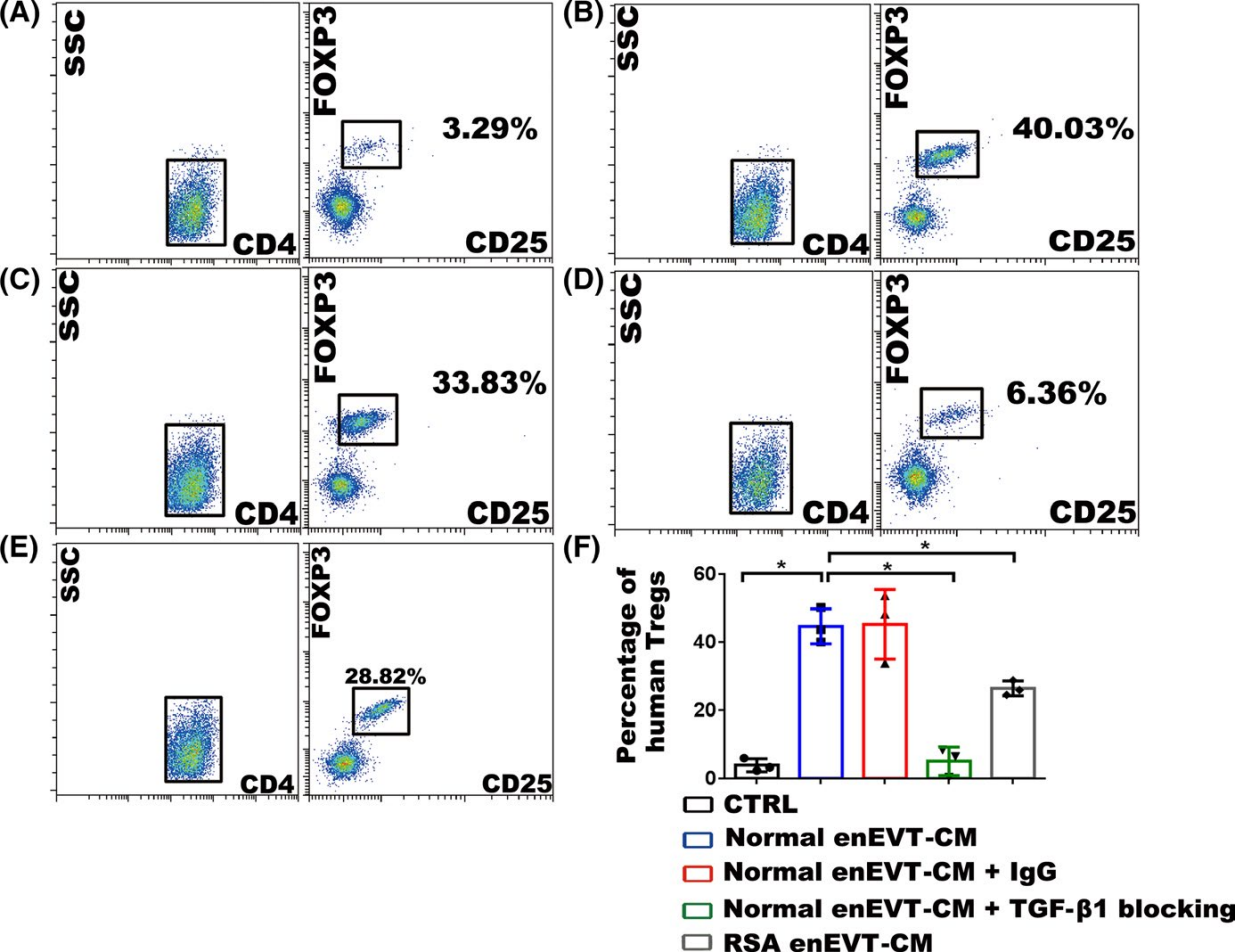
Effect of the conditioned media from enEVTs on the differentiation of Tregs. (A‐E) The results of flow cytometry showing the influence of the conditioned media from enEVTs (enEVT‐CM) on the differentiation of human Tregs. Human peripheral naïve CD4^+^ T cells are isolated and cultured in cell‐free RPMI‐1640 medium (CTRL; A), 50% RPMI‐1640 medium + 50% normal enEVT‐CM (B), 50% complete RPMI‐1640 medium + 50% normal enEVT‐CM + 20 μg/mL normal mouse IgG (C), 50% complete RPMI‐1640 medium + 50% normal enEVT‐CM + 20 μg/mL blocking antibody against TGF‐β1 (D), or 50% RPMI‐1640 medium + 50% RSA enEVT‐CM (E). The proportions of CD4^+^ CD25^+^ FOXP3^+^ Tregs were analysed after culture. F, Statistical analysis of flow cytometry showing the proportions of human CD4^+^ CD25^+^ FOXP3^+^ Tregs upon various treatments. The statistical analysis was performed based on the results from three independently repeated experiments using different batches of enEVT and naïve CD4^+^ T cells. Data are presented as mean ± SD, and the comparisons between groups were finally accomplished with Student's *t* test. *P < .05

Human naïve CD4^+^ T cells were isolated from the peripheral blood of healthy non‐pregnant women by using negatively selected magnetic sorting. Cell purity was more than 97%, as revealed by flow cytometry analysis for CD45RA and CD4 (Figure S3A). The purified human naïve CD4^+^ T cells were activated with anti‐CD3 and anti‐CD28 antibodies, followed by treatment with 50% enEVT‐CM for three days. The cells in the control group were treated with enEVT cell‐free–conditioned media. The percentage of CD4^+^ CD25^+^ FOXP3^+^ Tregs in enEVT‐CM–treated group was over 40% (Figure 4B), being approximately 13‐fold higher than that in control (Figure 4A,F). The enEVT‐CM was then pre‐incubated with the blocking antibody against TGF‐β1 (Figure 4D) or mouse IgG (Figure 4C) prior to treating naïve CD4^+^ T cells, and the effect of enEVT‐CM on inducing Treg differentiation was completely lost upon blocking TGF‐β1 (Figure 4C,D,F).

The experiment was conducted in parallel in mouse naïve CD4^+^ T cells. The CD4^+^ CD62L^+^ CD44^−^ mouse naïve T cells were sorted from virgin female mice (Figure S3B) and subjected to the treatment of enEVT‐CM with or without blocking antibody against TGF‐β1. It was shown that the mouse naïve CD4^+^ T cells could differentiate towards CD4^+^ CD25^+^ FOXP3^+^ Tregs upon enEVT‐CM treatment, and the portion of Tregs increased to approximately 10‐fold of that in the control group (Figure S4a,b,i). Blocking TGF‐β1 in enEVT‐CM could substantially eliminate the effect on inducing Treg differentiation (Figure S4c,d,i).

Because we found the production of TGF‐β1 in RSA enEVTs remarkably decreased, we proposed that RSA enEVTs might be less effective in inducing Treg differentiation. When human naïve CD4^+^ T cells were treated with the conditioned media from RSA enEVTs for 3 days, the proportion of Tregs was approximately 28%, which was <70% of that in the normal enEVT‐CM–treated group (Figure 4E,F).

**FIGURE 5:**
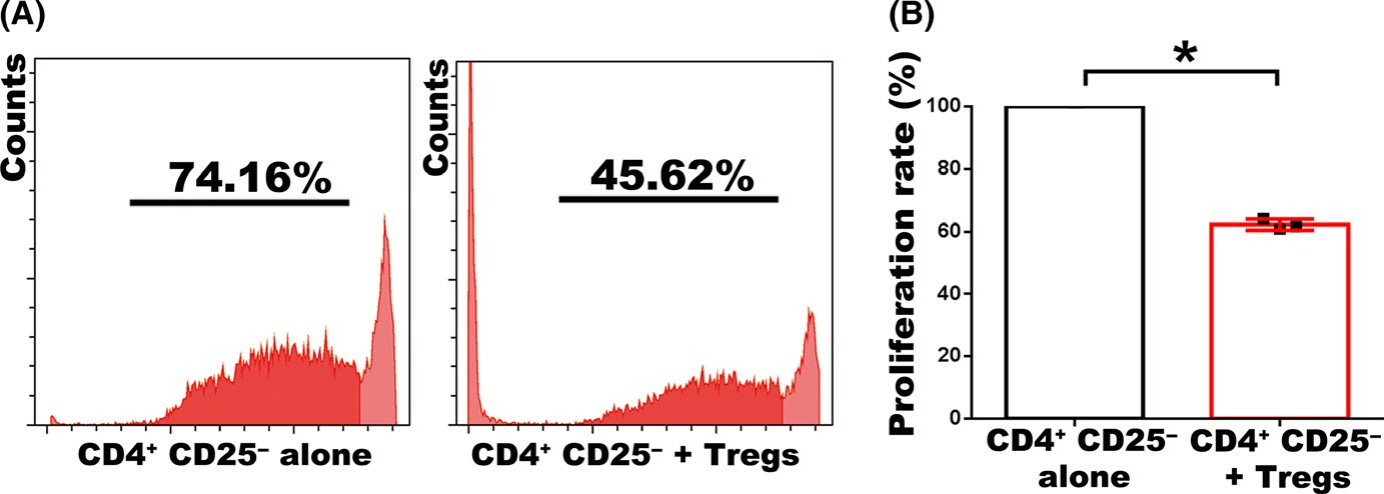
enEVT‐primed Tregs inhibit CD4^+^ CD25^‐^ T‐cell proliferation. A, The results of CFSE assay to measure the proliferation rate of CD4^+^ CD25^‐^ T cells that were co‐cultured with (right panel) or without (left panel) the enEVT‐primed Tregs. B, Statistical analysis of CFSE assay based on the results from three independently repeated experiments. Data are presented as Mean ± SD, and the comparison between groups was performed with Student's *t* test. *P < .05

To evaluate whether the enEVT‐primed Tregs are functional or not, we isolated the enEVT‐primed mouse Treg and co‐cultured with mouse CD4^+^ CD25^−^ T cells to observe T‐cell proliferation by CFSE assay. We found that CD4^+^ CD25^−^ T‐cell proliferation was significantly reduced by more than 45% after co‐culturing with the enEVT‐primed Tregs (Figure 5A,B), indicating that the enEVT‐primed Tregs were immunosuppressive.

### 3.4 Neither iEVTs nor dECs could induce differentiation of Tregs

We cultured the primary iEVTs and dECs and collected their conditioned media (iEVT‐CM and dEC‐CM) at 24 hours of culture. Either human or mouse naïve CD4^+^ T cells were treated with 50% iEVT‐CM or dEC‐CM for three days. As shown, neither iEVT‐CM (Figure S4e,g) nor dEC‐CM (Figure S4f,h) had any effect on human or mouse T‐cell differentiation towards CD4^+^ CD25^+^ FOXP3^+^ Tregs (Figure S4i,j).

The results indicated that enEVTs were functionally different from iEVTs and dECs in inducing differentiation of Tregs.

## 4 DISCUSSION

**FIGURE 6:**
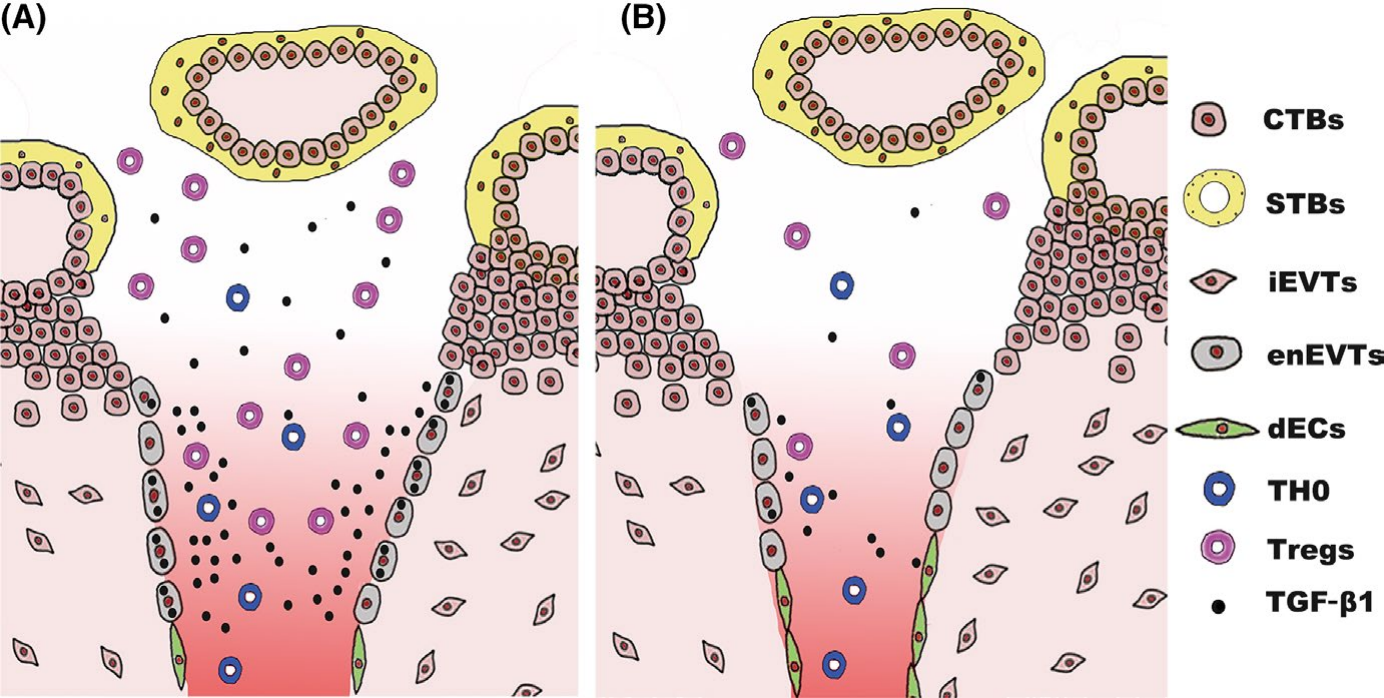
A scheme of the suggested model based on the results revealed in this study. During normal pregnancy (A), enEVTs replace the dECs and produce TGF‐β1 to educate maternal CD4^+^ T‐cell differentiation to Tregs when maternal blood perfuses through the remodelled SPA. The expanding Tregs along the placental‐maternal circulation contribute substantially to form a safe microenvironment in the remodelled SPA and IVS, which protects enEVTs and villous trophoblasts from maternal immune attack. However, in RSA patients (B), the total number of enEVTs and the ability to produce TGF‐β1 in enEVTs decline sharply. The proportion of Tregs decreases accordingly in SPA and IVS, which may lead to the immune attack of trophoblasts by maternal lymphocytes and therefore the adverse pregnancy outcome

The establishment of an immune‐tolerant environment at the maternal‐foetal interface has been proven as a result of the complex interaction among various cell types. Numerous studies indicated the direct or indirect crosstalk between EVTs and the maternal immune cells in the decidua. For instance, iEVTs express human leucocyte antigen (HLA) class I ligands to interact with the inhibitory receptor on decidual natural killer cells (dNKs) and repress the cytotoxicity of dNKs.^39^, ^40^, ^41^ The Th2‐bias cytokines secreted by dNKs, M2 macrophages and Th2 cells facilitate the invasion ability of EVTs.^42^, ^43^ Besides the decidua part, there are other interfaces at risk of immune rejection, primarily including the remodelled SPA and IVS where the placental‐maternal circulation is generalized. In the remodelled SPA, enEVTs come in direct contact with maternal lymphocytes. In the IVS area, foetal villous trophoblasts immerse into maternal blood that perfuses from SPA. However, it remains unclear how immune tolerance is established along this placental‐maternal circulation pathway. Our present study reveals the unique contribution of enEVTs to immune tolerance along this pathway. As we summarize in Figure 6A, enEVTs in the remodelled SPA, but not iEVTs or dECs, predominantly produce TGF‐β1, which actively induces maternal naïve CD4^+^ T‐cell differentiation towards immunosuppressive FOXP3^+^ Tregs when maternal blood flows through the remodelled SPA. The booming Tregs, therefore, contribute essentially to forming safe microenvironment in the remodelled SPA and IVS, where enEVTs and villous trophoblasts are protected from maternal immune attack. In RSA cases, the enEVTs are less in number and exhibit significantly limited potential to produce TGF‐β1. In parallel, the portions of FOXP3^+^ Tregs in remodelled SPA and IVS are substantially reduced compared with the healthy pregnant controls (Figure 6B). Significant decline of Treg amount in RSA decidua and peripheral blood, as well as the diminishment of circulating TGF‐β1 and IL‐2 in RSA patients, has been demonstrated in many other studies.^19^, ^20^, ^21^, ^26^, ^27^, ^28^ Thus, it is possible that the insufficient immune tolerance in RSA pregnancy may, at least in part, result from the dysregulation of functional enEVT differentiation.

The interaction between enEVTs and Tregs, as revealed in this study, provides novel evidence to understand why the uterine endothelial cells need to be replaced by enEVTs in the remodelled SPA. It has been believed that iEVTs and enEVTs function together to make the decidual spiral arteries less resistant and of higher capacity. However, the properties of enEVTs have not been well recognized. Evidence indicated the specific expression of VE‐Cadherin, NCAM1 and Jagged1 in enEVTs. In addition, our data revealed the strong and specific ability of enEVTs to produce TGF‐β1. In vitro co‐culture study by Tilburgs et al^44^ showed that EVTs can directly enhance the proportion of FOXP3^+^ Tregs, and our study further demonstrated that only enEVTs but not iEVTs had this potential. Tilburgs et al assumed that the activation of Notch1 signalling in CD4^+^ T cells by Notch1 ligands (Jagged1 and DDL1) in mouse dECs might facilitate the differentiation of T cells towards Tregs.^45^ Interestingly, enEVTs can extensively express Jagged1,^46^ which indicates their potential to interact with CD4^+^ T cells through Notch signalling. We suggest that enEVTs sustain multiple and possibly redundant mechanisms to guarantee maternal Treg differentiation and thus create a tolerogenic immune environment along the placental‐maternal circulation. In addition, it is well known that TGFβ is a potent regulator of immune cells. It can convert CD16^+^ peripheral natural killer cells (pNKs) into CD16^‐^ NK cells with similarity to dNK phenotype.^47^ We assume that enEVTs may also participate in educating pNKs into dNKs by producing TGFβ1 and other cytokines along the placental‐maternal circulation. These evidences convincingly explain the physiological significance of replacing the spiral artery endothelial cells by enEVTs from the aspect of immune tolerance. Further characterization of enEVTs in regulating immune cell composition at the maternal‐foetal interface is of key importance for understanding the pathogenesis of severe pregnancy disorders such as RSA and preeclampsia.

It has been suggested that the increase in Tregs in decidual tissue was due to the local expansion or a selective recruitment of Tregs to the maternal‐foetal interface.^48^, ^49^ From our immunofluorescence results, Tregs predominantly existed in remodelled SPA and IVS, while were much less in the decidual stroma and rare in unremodelled SPA. The data supported the idea of the selective enrolment and education of peripheral T cells through the placental‐maternal circulation pathway, where enEVTs play critical roles. Furthermore, maternal T cells in decidual vessels might transmigrate across the vascular endothelial cell layer through the activation of Notch signalling in T cells by Jagged1 or DDL1 in dEC, and differentiate to Tregs in response to TGFβ produced in decidual macrophages (dMɸ) and decidual stromal cells.^45^ To fully clarify the physiological mechanism, studies in optimal animal models are necessary. Gene manipulation in invasive trophoblasts or endothelial cells at different stages of the spiral artery remodelling may provide further insights into the contribution of enEVT infiltration to the unique dynamics of leucocyte differentiation in decidual tissue during pregnancy.

An interesting observation in this study is that around 25% of enEVTs are TGF‐β1–positive, indicating the heterogeneous subtypes of enEVTs. Simple histological observation showed different localization of enEVTs, either attaching to the vessel wall or floating in the lumen of SPA (Figure S1A). It has been suggested that enEVTs floating in the lumen may function to reduce the blood flow velocity.^50^, ^51^, ^52^ We did not find any convincing correlation between TGFβ1 expression in enEVTs and their localization. Further investigation is needed to clarify the subtype properties of enEVTs, which will help to expand our understanding of pregnancy maintenance, as well as the aetiology of pregnancy disorders, such as RSA and preeclampsia.

It has been reported that the spiral artery remodelling is insufficient in RSA patients,^53^ in line with our observations that the number of remodelled SPA and enEVTs in SPA significantly reduced in RSA decidua. In addition, the proportion of TGF‐β1–producing enEVTs and the production of TGF‐β1 in enEVTs were much less in RSA decidua. This is in parallel with the evidence that circulating TGF‐β1 concentration in RSA cases was significantly lower than normal pregnant controls.^26^, ^27^, ^28^, ^29^ Interestingly, TGF‐β1 can promote SPA remodelling through inducing HIF‐1α expression and subsequently stimulating VEGF expression in trophoblasts.^54^, ^55^ It is feasible to suggest that the reduction in TGF‐β1 in RSA decidua may, at least partially, lead to insufficient SPA remodelling, which further impairs the immune cell differentiation. Therefore, the multiple roles of TGF‐β1 in regulating trophoblast function and maintaining immune tolerance indicate its central character in maternal‐foetal communication.

In summary, the findings in this study demonstrate a unique immune‐regulatory characteristic of placental enEVTs to educate maternal CD4^+^ T‐cell differentiation into Tregs. This is an important cellular mechanism to develop an immune‐tolerant environment along the placental‐maternal circulation pathway. The study also provides new insights into revealing the mechanisms of immune imbalance in the development of pregnancy complications such as RSA.

## CONFLICT OF INTEREST

The authors declare no competing interests.

## AUTHOR CONTRIBUTIONS

YM performed the experiments, interpreted the data and drafted the manuscript. QY and MF collected clinical samples, analysed clinical data and interpreted the data. WJ, ZL, FW and YL participated in analysing the data. LZ, YG, JW and RL collected clinical samples and helped in analysing clinical data. YW and XS designed and supervised the study, and revised the manuscript.

## Supporting information

Supplementary Material

## Data Availability

The data of this study are available from the corresponding author upon reasonable request.
