# Supplementary material for: Placental endovascular extravillous trophoblasts (enEVTs) educate maternal T‐cell differentiation along the maternal‐placental circulation
Source: Cell Prolif. 2020 Apr 14;53(5):e12802. doi: 10.1111/cpr.12802 (PMC7260064; doi:10.1111/cpr.12802)
Supplement: Supplementary file 1 — Supplementary Material [file CPR-53-e12802-s001.docx]

**Supplementary Table S1**

The cases of RSA patients.

| **Patient**  **Number** | **Age (y)** | **Gestational**  **Age (d)** | **History of Previous Miscarriages** |
| --- | --- | --- | --- |
| **1** | **37** | **56** | **4** |
| **2** | **40** | **60** | **4** |
| **3** | **35** | **62** | **3** |
| **4** | **32** | **53** | **3** |
| **5** | **28** | **52** | **3** |
| **6** | **32** | **49** | **2** |
| **7** | **33** | **56** | **3** |
| **8** | **35** | **55** | **3** |
| **9** | **29** | **63** | **2** |
| **10** | **42** | **77** | **3** |

**
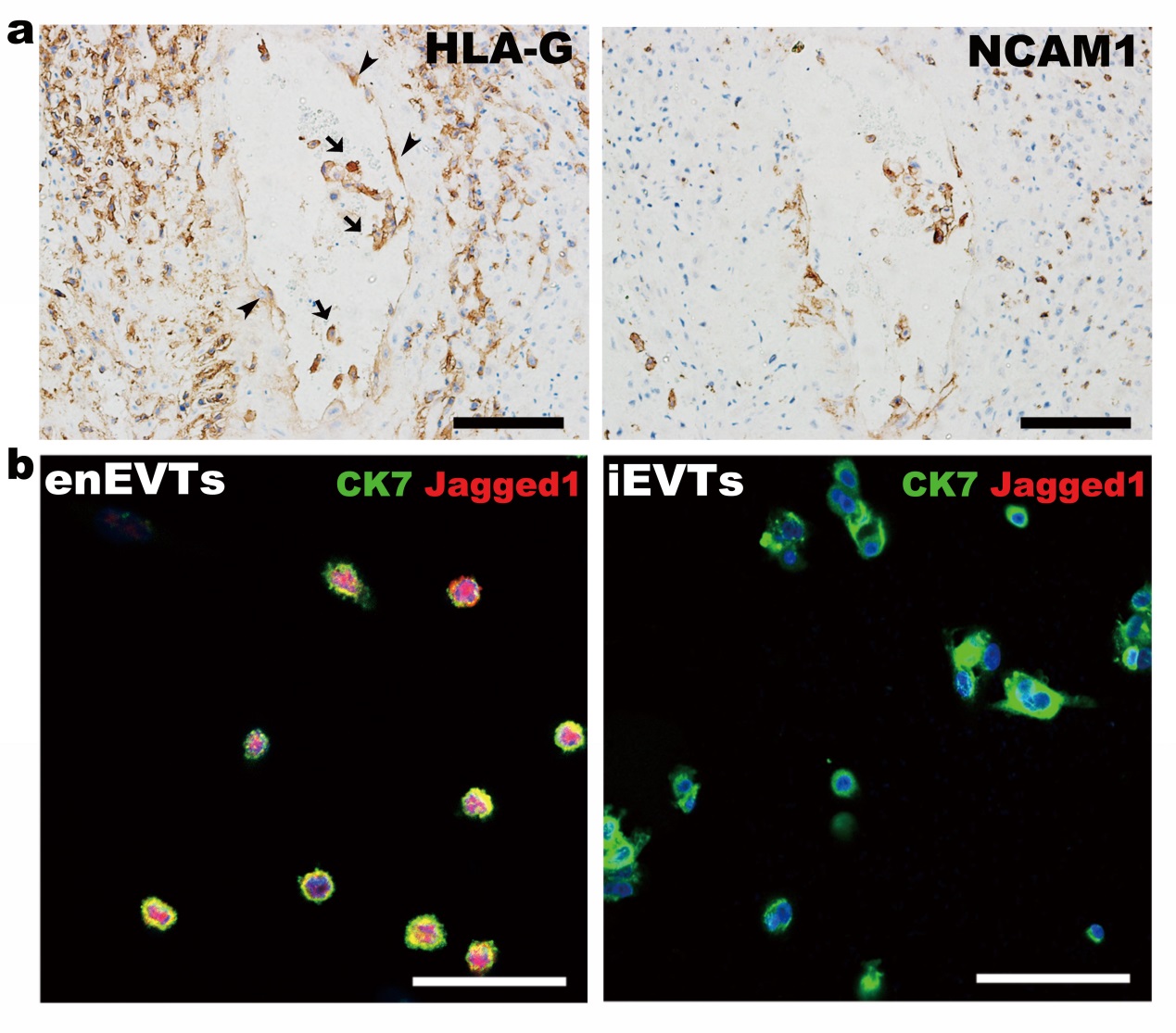
**

**Supplementary Figure S1**

**a** Immunohistochemistry staining of HLA-G (Left panel) and NCAM1 (Right panel) in enEVTs. Arrows indicate the enEVTs float inside the SPA lumen. Arrow heads indicate the enEVTs attach to the SPA lumen. **b** Immunofluorescent staining of CK7 (green) and Jagged1 (red) in FACS isolated enEVTs (Left panel) and iEVTs (Right panel). Scale bars= 50μm.


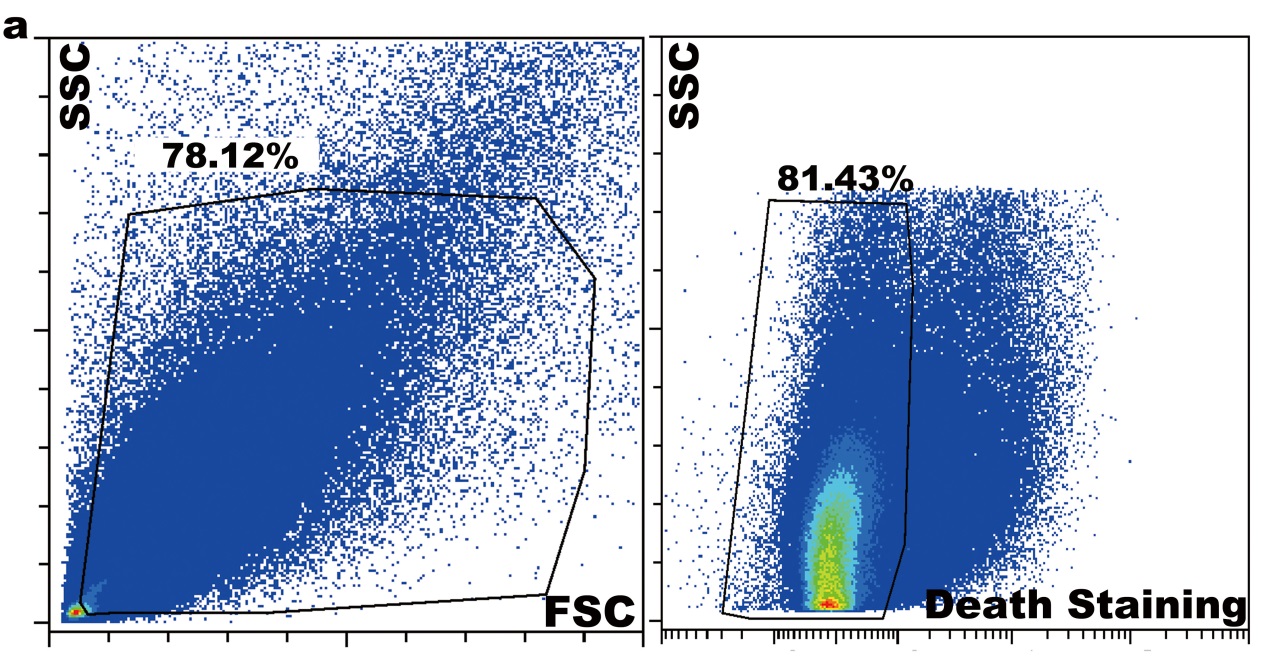


**Supplementary Figure S2**

FACS isolation of living primary cells from normal early pregnant decidua.


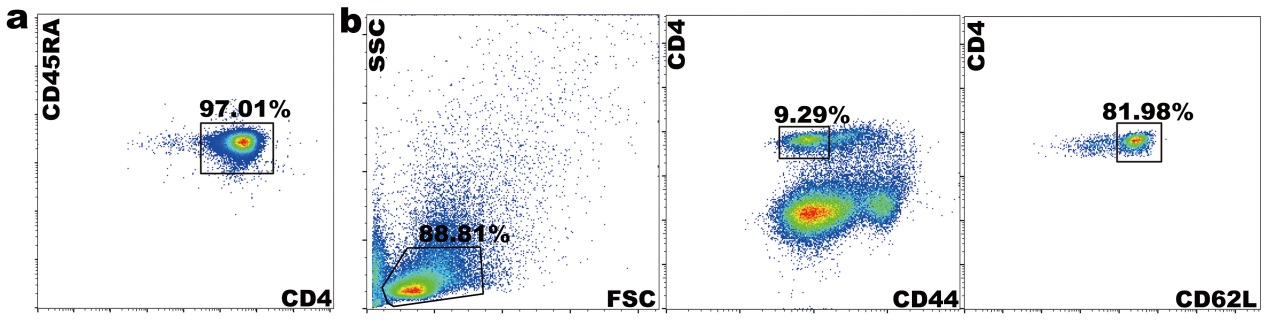


**Supplementary Figure S3**

**a** Flow cytometry analysis of MACS isolated human peripheral Naïve CD4^+^ T cells with antibodies against CD4 and CD45RA. **b** FACS isolation of mice Naïve CD4^+^ T cells gated from CD4^+^ CD62L^+^ CD44^-^.

**
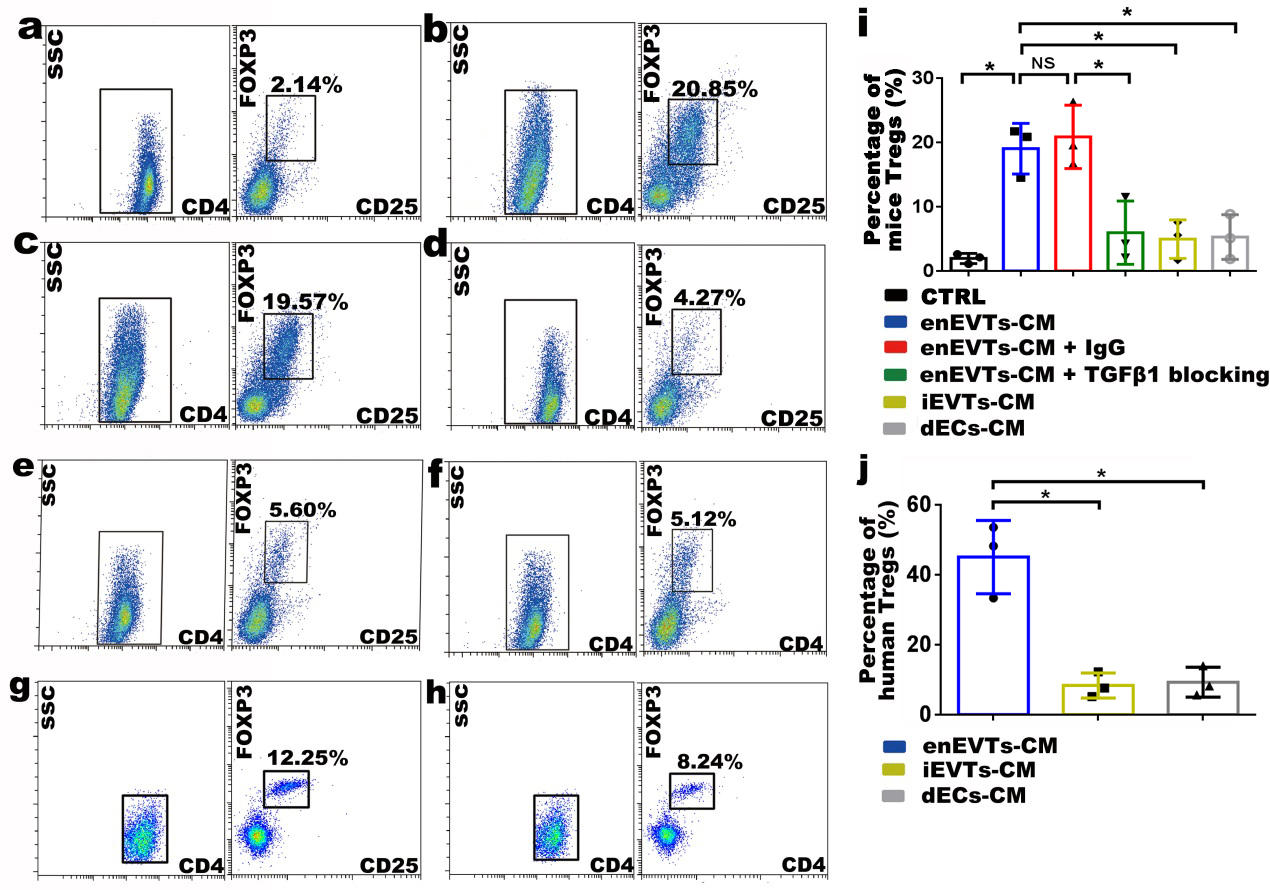
**

**Supplementary Figure S4**

**Effect of the conditioned media from enEVTs on the differentiation of mice Tregs. iEVTs and dECs had little function on Tregs differentiation. a-f** The results of flow cytometry showing the influence of the conditioned media from enEVTs (enEVTs-CM) on the differentiation of mice Tregs. Mice naïve CD4^+^ T cells are isolated and cultured in cell-free RPMI-1640 medium (CTRL; a), 50% RPMI-1640 medium + 50% enEVTs-CM (b), 50% complete RPMI-1640 medium + 50% enEVTs-CM + 20μg/ml normal mouse IgG (c), 50% complete RPMI-1640 medium + 50% enEVTs-CM + 20μg/ml blocking antibody against TGFβ1 (d), 50% RPMI-1640 medium + 50% iEVTs-CM (e) or 50% RPMI-1640 medium + 50% dECs-CM (f) . The proportions of CD4^+^ CD25^+^ FOXP3^+^ Tregs were analyzed after culture. **g-h** The results of flow cytometry showing the influence of the conditioned media from iEVTs-CM and dECs-CM on the differentiation of human Tregs. Human peripheral naïve CD4^+^ T cells are cultured in 50% RPMI-1640 medium + 50% iEVTs-CM (g) or 50% RPMI-1640 medium + 50% dECs-CM (h). The proportions of mice CD4^+^ CD25^+^ FOXP3^+^ Tregs are analyzed after culture. **i-j** Statistical analysis of flow cytometry showing the proportions of mice (i) or human (j) CD4^+^ CD25^+^ FOXP3^+^ Tregs upon various treatments. The statistical analysis was performed based on the results from three independently repeated experiments. Data are presented as mean±SD, and the comparisons between groups were finally accomplished with Student's t-test. *, P < 0.05. NS, no significance.
